# Female Cuckoo Calls Are More Effective at Attracting Breeding Male Cuckoos and Their Hosts Than Male Cuckoo Calls

**DOI:** 10.1002/ece3.72069

**Published:** 2025-08-27

**Authors:** Hanlin Yan, Wei Liang

**Affiliations:** ^1^ School of Biological Science and Technology Liupanshui Normal University Liupanshui China; ^2^ Ministry of Education Key Laboratory for Ecology of Tropical Islands, Key Laboratory of Tropical Animal and Plant Ecology of Hainan Province, College of Life Sciences Hainan Normal University Haikou China

**Keywords:** brood parasitism, common cuckoo, female call, host, playback experiment

## Abstract

Vocalizations during the breeding season play a significant role in both intra‐ and inter‐specific communications. There have been numerous studies on the calls and functions of the common cuckoo ( *Cuculus canorus* [CC]). However, there is limited research on the calls and functions of female CCs. This study conducted a playback experiment of 90 sample sizes on actively breeding CCs and their hosts in Jilin Province, China, during the 2022 breeding season (May to August), with calls from female CCs, male CCs, and males of the Himalayan cuckoo (*C. saturates* [CS]) as control groups. The results showed that the calls of both male and female CCs attracted CCs and their hosts in the experimental area, while the control group did not attract any birds. The attracted CCs were predominantly males. Among the attracted hosts, there were Oriental reed warblers ( *Acrocephalus orientalis* ) and black‐browed reed warblers ( *Acrocephalus bistrigiceps* ). Generalized linear mixed model analysis revealed that the number of individuals attracted by female CC calls was significantly higher than the number attracted by male CC calls, both for male CCs and hosts. Furthermore, male CCs were attracted by male and female CC calls to a significantly greater extent during the early stage than during the late stage, while there was no difference in the number of attracted host individuals. Our study showed that the calls of female CCs play an important role in the communication between male and female CCs, and hosts are more responsive to the calls of female CCs, suggesting that they perceive female cuckoos as more threatening. Our study reveals seasonally modulated male attraction and host sensitivity.

## Graphical abstract

Vocalizations during the breeding season play a significant role in both intra‐ and interspecific communications. The number of individuals attracted by female common cuckoo ( *Cuculus canorus* ) calls was significantly higher than the number attracted by male calls, both for male cuckoos and hosts. Our study showed that female cuckoo calls play an important role in the communication between male and female cuckoos, and hosts are more responsive to female cuckoo calls, suggesting that hosts perceive female cuckoos as more threatening.

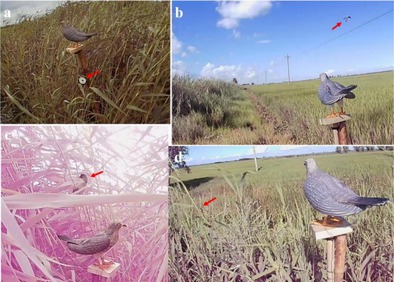

## 1 Introduction

Auditory communication in animals evolved as a method of information exchange that has developed over a long period. This type of communication allows information to be sent, received, and utilized between conspecifics or individuals of different species (Maynard‐Smith and Harper 2003; Bradbury and Vehrencamp 2011). Animal vocalizations are closely linked to their behavioral contexts, such as mate attraction, alarm calls and mobbing, group foraging, and nocturnal roosting (Marler 2004). Among animals, birds have evolved highly sensitive and sophisticated vocal signals, which serve as an effective means of long‐distance communication, especially in environments with visual obstructions (Catchpole and Slater 2008). For example, songbirds with diverse repertoires may share some song types with their neighbors, aiding in mutual recognition (Briefer et al. 2008; Stoddard 1996). However, songbirds with limited and simple repertoires may rely on distinctive acoustic features for discrimination and identification (Osiejuk 2014).

The common cuckoo ( *Cuculus canorus* [CC]) is an obligate avian brood parasite that lays its eggs in the nests of other bird species, known as hosts. This behavior forces the host to incubate the parasitic eggs and feed the cuckoo nestlings at the expense of their own reproduction (Wyllie 1981; Davies 2000). The CC is known for its simple dual‐note “cu‐coo” call, which is continuously produced by male CCs at their breeding sites (Lei et al. 2005; Moskát and Hauber 2022). However, recent research indicates that syntactic errors of male CCs, including syllable tripling (e.g., “cu‐cu‐coo”), can occur in these typically stereotypical calls and influence host responses (Tryjanowski, Jankowiak, Indykiewicz, et al. 2025). Conversely, female CCs rarely vocalize and use a so‐called “bubbling” call during the breeding season, which may serve various communicative functions (Deng et al. 2019; Moskát and Hauber 2019, 2022, 2023; Yoo et al. 2020; Jin et al. 2025). For example, within conspecifics, female CCs use their “bubbling” calls to signal their chosen laying site, reducing aggression between females (Moskát and Hauber 2019; Moskát et al. 2020; Xia et al. 2019), attracting mates (Moskát and Hauber 2019, 2023; Xia et al. 2019), or engaging in duets with males (Moskát and Hauber 2021; Hauber and Moskát 2025).

The calls emitted by CCs not only play a crucial role in conspecific communication but also serve as an effective strategy to enhance reproductive success (Moskát et al. 2017, 2020; Moskát and Hauber 2019; Yu et al. 2019). Several studies have reported on the vocalizations of CCs (Deng et al. 2019; Wang et al. 2021; Moskát and Hauber 2021, 2022; Moskát and Hauber 2021; Elek et al. 2021; Tryjanowski et al. 2024). For example, CCs can distinguish between the calls of neighbors (Moskát et al. 2017), and female CC calls have been shown to induce anti‐predator behavior in birds (Zhang et al. 2021) and elicit vigilance and avoidance responses in wild free‐range chickens (Jiang et al. 2021). Furthermore, female CC calls influence the behavior of their hosts (York and Davies 2017; Wang et al. 2022). Marton et al. (2021) even demonstrated that female CC calls can suppress host aggression. However, to our knowledge, there has been no research regarding the calls of female CCs and their functions (but see Hauber and Moskát 2025; Jin et al. 2025), especially in regard to intra‐specific communication.

While male cuckoo calls have been extensively studied for their role in territoriality and conspecific communication (Moskát and Hauber 2022; Tryjanowski et al. 2024), the functions of female‐specific ‘bubbling’ calls remain less understood, particularly regarding their dual role in intra‐ versus inter‐specific interactions. Recent evidence suggests female calls may simultaneously attract mates (Moskát and Hauber 2019; Hauber and Moskát 2025) and manipulate host behavior (Marton et al. 2021; York 2021), but comparative efficacy data are lacking. In this study, we designed playback experiments that involved playing the calls of male and female CCs and males of the Himalayan cuckoo (*Cuculus saturates* [CS]) as a control during the breeding season to assess the behavioral responses of CCs or hosts in the experimental area to these three types of sounds. We hypothesized that the female CC calls will attract more conspecific males than male calls, reflecting their role in sexual signaling. And female CC calls will elicit stronger host responses than male calls, as hosts associate them with imminent parasitism risk.

## 2 Materials and Methods

### 2.1 Study Area and Study Species

The Sifangtuozi Farm (46°00′–46°22′ N, 123°46′–123°57′ E) is located in Zhenlai County, in the Nenjiang River basin, a tributary of the Songhua River, in the northeastern Jilin Province, China. The area is characterized by abundant water resources, and extensive stands of cattails and reeds can be found in various areas surrounding the farm, including field dikes, irrigation channels, streams, and ponds. Every breeding season (May–August), many individuals of the Oriental reed warbler ( *Acrocephalus orientalis* [ORW]) and black‐browed reed warbler ( *Acrocephalus bistrigiceps* [BRW]) migrate to these wetlands to breed (Trnka et al. 2023). ORWs are the primary hosts of CCs in Asia. In northern China, the parasitism rate of the CCs can reach as high as 34.3% to 65.5% (Yang et al. 2016, 2017).

Between June and August 2022, we located 26 BRW nests and 123 ORW nests, with a total of 38 ORW nests being parasitized, resulting in a cuckoo parasitism rate of 30.8%. Given that the ORW is a primary host for local CCs, there has been a high degree of coevolution between them (Wang et al. 2022). Conversely, the BRW is a rare host for CCs (Yang et al. 2022), with only one parasitized nest found in the Zhalong area of Heilongjiang, China, resulting in a parasitism rate of 0.42% (Yang et al. 2017). In the study area, during the breeding season, a significant number of CCs were observed perching on branches of reed beds, utility poles, and power lines around the reed beds.

### 2.2 Field Experiments

We conducted experiments during both the early and late stages of the host, because they represent distinct phases of parasitism risk: “early stage (May–June)” coincides with peak cuckoo egg‐laying, while “late stage (July–August)” represents a period when parasitic events typically cease almost entirely. Playback experiments were conducted using female and male CC calls as the experimental group, with CS calls used as the control group. Each of the three types of calls was tested 15 times during both the early and late stages: female CC (early stage, *n* = 15; late stage, *n* = 15), male CC (early stage, *n* = 15; late stage, *n* = 15), and CS (early stage, *n* = 15; late stage, *n* = 15). Each experiment was conducted at randomly selected points within the reed bed, with a minimum distance of 200 m between each experimental point, and no point was repeated. To minimize the impact of environmental noise on sound playback, experiments were scheduled to avoid agricultural machinery operations, roadside traffic, and noisy conditions during strong winds or rainy weather. The period of time we conducted the experimental test was random on each day (from sunrise to sunset), specific time frames were not designated (e.g., morning, afternoon, 8 a.m.–1 p.m. or similar). For each sampling point, we conducted sound playback without prior knowledge of the presence of host nests nearby. Sound playback was conducted at each sampling point regardless of the presence or absence of CCs.

To reduce the potential effects of pseudoreplication, we prepared two 3D‐printed models of gray CCs (referred to as 3D dummies) in advance (Trnka et al. 2023). We prepared two sound recordings of female CCs, male CCs, and male CSs, all obtained from Xeno Canto recordists (https://xeno‐canto.org/). We constructed playlists with a 22,050 Hz sampling rate and 16‐bit sampling format and normalized peak amplitude at −3 dB, which can be found in the Audios [Link], [Link]. In each trial, sound playback experiments were conducted at the maximum volume of the playback device (85 db measured at 1 m distance from the speaker).

**FIGURE 1:**
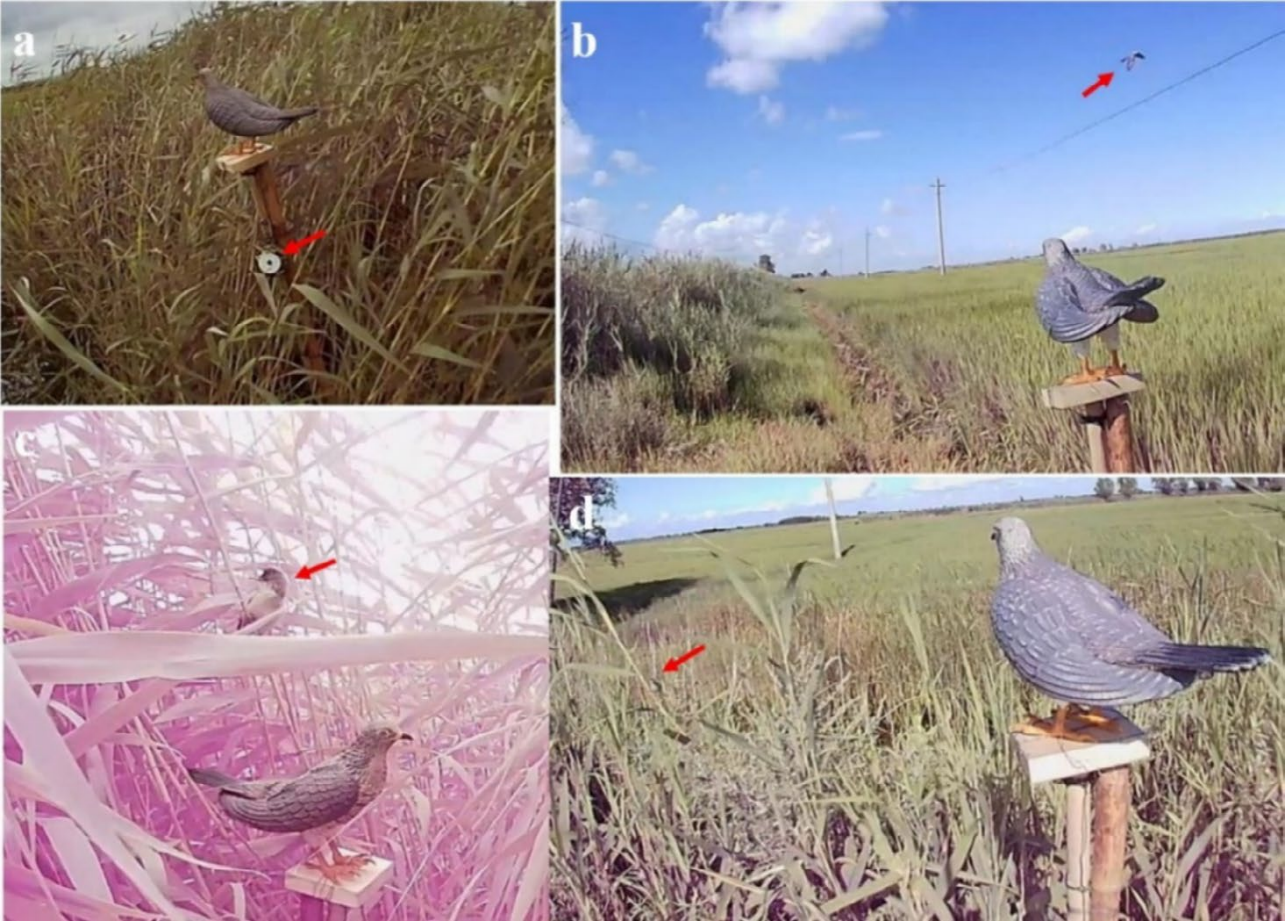
Setting during sound playback [red arrow in (a) indicates loudspeaker, red arrow in (b) indicates common cuckoo approaching the 3D model in flight during sound playback, red arrow in (c) indicates Oriental reed warbler approaching the 3D model, and red arrow in (d) indicates black‐browed reed warbler approaching the 3D model].

During each experiment, a random combination of 3D dummies and sound was selected for on‐site testing, with each sound played for a duration of 2 min. We displayed the 3D dummies of gray CCs (Figure 1) during sound playback. To facilitate the recording of the experimental setup, we prepared a display stand of approximately 2 m in height. During experiments, the 3D dummies were placed on the display stand, and a Bluetooth speaker (See Me Here E1; Shenzhen See Me Here Electronic Co. Ltd., Shenzhen, China) was positioned underneath the dummy display stand (Figure 1a). A video recorder (Ou Chuang A8; Xiamen Shangyu Huajin Electronic Technology Co. Ltd., Xiamen, China) was placed at ca. 2 m from the dummy display stand to record real‐time conditions during sound playback. To prevent observer bias, the sound type played during each trial was concealed from field observers. Video recordings were subsequently analyzed by two independent researchers blinded to the treatment groups.

### 2.3 Statistical Analyses

All data were statistically analyzed using IBM SPSS Statistics 25. All tests were two‐tailed, and a significance level of *p* < 0.05 was used. Generalized linear mixed models (GLMMs) were used to compare the responses of CCs (Figure 1b) in their habitat to the three calls during both the early and late stages. The responses of the hosts, BRW and ORW (Figure 1c,d), to the calls were also analyzed.

We recorded the behavioral responses of CCs or hosts near the dummies during sound playback as 1 (such as approaching the dummy from a distance and perching on nearby branches, flying and circling above the dummy, or flying and attacking the dummy). Behaviors that did not involve approaching (e.g., no birds approaching, staying in place while vocalizing, or fleeing) were recorded as 0. Sampling points ID were considered as the random effect, time as the repeated measure, and the breeding stage (early and late stages) and sound types (female CC, male CC, and male CS) were considered as fixed effects. Pairwise comparison of behavioral responses between the early and late stages was conducted. The male CS playback data were excluded from the GLMM analysis because no behavioral responses (all recorded as 0) were observed across all 90 trials. This absence of variation precluded meaningful statistical comparison with the experimental groups (female and male CC calls), which showed significant responses. We performed pairwise comparisons between the female and male CC calls within the sound types. The main objectives of the analysis were to compare the number of sampling points where CCs approached the dummies, the number of individual CCs that approached the dummies, the distance of CCs approaching the dummies, the number of sampling points where hosts (the sum of ORW and BRW) approached the dummies, the number of sampling points where ORWs approached the dummies, the number of sampling points where BRWs approached the dummies, the number of host individuals approaching the dummies, the number of ORWs approaching the dummies, and the number of BRWs approaching the dummies.

## 3 Results

### 3.1 Common Cuckoos Approaching the Dummy During Sound Playback

Overall, the playback of male CS calls did not elicit any response from CCs and their hosts during both early and late stages. There was a total of 27 sampling points (out of 90) where CCs approached the dummies during sound playback (Table 1). Although the number of sampling points where CCs approached the dummies varied among the two breeding stages and the three sound playback conditions, GLMM analysis revealed a significant difference in the number of sampling points where CCs approached between early and late stages (*p <* 0.001). Additionally, there were significantly more sampling points where female CC calls attracted CCs than points where male CC calls attracted them (*p* = 0.001).

**TABLE 1:** Number of sampled points with CCs or hosts approaching the dummy during sound playback.

	Early stage			Late stage		
	Female CC	Male CC	CS	Female CC	Male CC	CS
Points of CCs	11	7	0	6	3	0
Points of hosts	8	2	0	5	0	0
Points of ORWs	3	0	0	2	0	0
Points of BRWs	5	2	0	3	0	0
Sampled points	15	15	15	15	15	15

Abbreviations: BRW, black‐browed reed warbler; CC, common cuckoo; CS, the Himalayan cuckoo; ORW, Oriental reed warbler.

**FIGURE 2:**
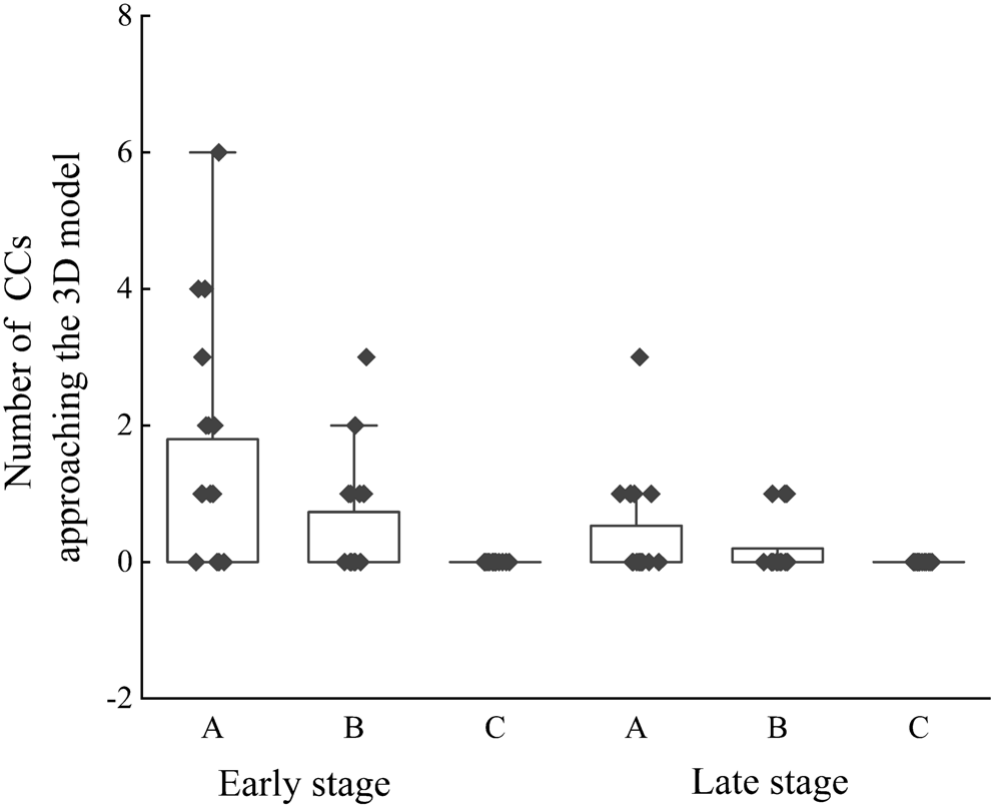
Number of common cuckoos (CCs) approaching the 3D model (A refers to female CC calls, B refers to male CC calls, and C refers to male Himalayan cuckoo calls).

During sound playback, 49 CCs approached the dummies (Figure 2; Table 2). Those attracted by the sound playback were only male CCs. The number of male CCs attracted by the female CC calls (35 in total) was significantly higher than that attracted by male CC calls (14 in total) (*p* = 0.003, GLMM). Additionally, significantly more male CCs were attracted during the early stage (38 in total) than during the late stage (11 in total) (*p <* 0.001, GLMM) (Table 3).

**TABLE 2:** Number of CCs or hosts approaching the dummy during sound playback.

	Early stage			Late stage		
	Female CC	Male CC	CS	Female CC	Male CC	CS
Number of CC	27	11	0	8	3	0
Number of hosts	10	2	0	6	0	0
Number of ORWs	3	0	0	2	0	0
Number of BRWs	7	2	0	4	0	0

*Note:* “Host” is the sum of ORW and BRW.

Abbreviations: BRW, black‐browed reed warbler; CC, common cuckoo; CS, the Himalayan cuckoo; ORW, Oriental reed warbler.

**TABLE 3:** Results of generalized linear mixed model.

	Breeding stage (early vs. late stages)				Sound type (female vs. male CCs)			
	Coefficient	SD	*t*	*p*	Coefficient	SD	*t*	*p*
Number of sample points CC	0.450	0.104	4.312	**< 0.001**	0.355	0.106	3.348	**0.001**
Number of CC	0.798	0.188	4.252	**< 0.001**	0.410	0.133	3.084	**0.003**
CC approaching distance	2.275	0.765	2.975	**0.004**	1.750	0.855	2.048	**0.045**
Number of sample points H	0.000	0.000	0.004	0.997	0.370	0.084	4.403	**< 0.001**
Number of sample points ORW	0.000	0.000	0.003	0.998	0.000	0.000	0.004	0.997
Number of sample points BRW	0.000	0.000	0.005	0.996	0.210	0.071	2.963	**0.004**
Number of hosts	0.000	0.000	0.003	0.998	0.430	0.109	3.965	**< 0.001**
Number of ORW	0.000	0.000	0.003	0.998	0.000	0.000	0.004	0.997
Number of BRW	0.000	0.000	0.003	0.997	0.205	0.085	2.427	**0.018**

*Note:* “Number of sample points CC” represents the number of sample points where common cuckoos approached the 3D model. “Number of CC” represents the number of common cuckoos that approached the 3D model. “CC approaching distance” represents the distance at which common cuckoos approached the 3D model. “Number of sample points H” represents the number of sample points where hosts approached the 3D model. “Number of H” represents the number of hosts that approached the 3D model. “Number of sample points ORW” represents the number of sample points where Oriental reed warblers approached the 3D model. “Number of sample points BRW” represents the number of sample points where black‐browed reed warblers approached the 3D model. “Number of ORW” represents the number of oriental reed warblers that approached the 3D model. “Number of BRW” represents the number of black‐browed reed warblers that approached the 3D model. “Host” represents the sum of Oriental reed warblers and black‐browed reed warblers. Significant effects (*p* < 0.05) are indicated in bold.

**FIGURE 3:**
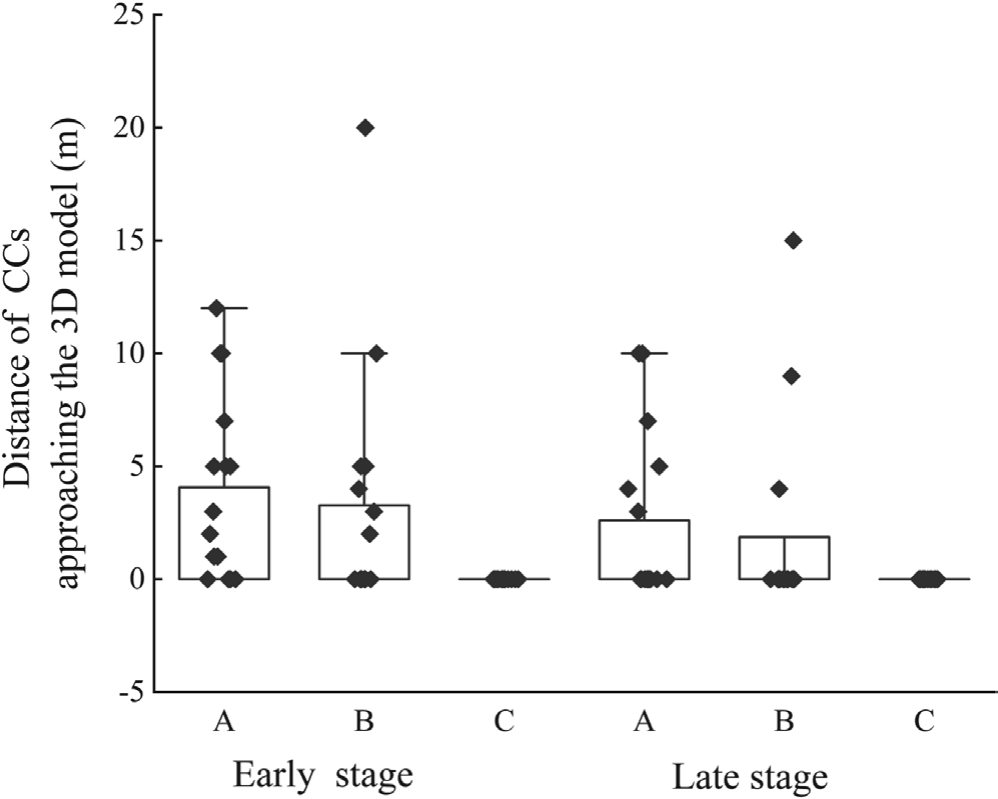
Distance of common cuckoos (CCs) approaching the 3D model (A refers to female CC calls, B refers to male CC calls, and C refers to male Himalayan cuckoo calls).

Among all the points where CCs approached the dummies, the distance at which each CC approached the dummies varied (mean distance ± SD, 6.55 ± 4.48 m, range 1–20 m; *n* = 27) (Figure 3). CCs attracted by the sound playback approached closer to the dummies during the early stage than during the late stage. Moreover, when comparing female CC calls to male CC calls, the CCs approached more closely to the dummies. The GLMM analysis indicated that the distance at which these CCs approached the dummies was significantly different, both in terms of the breeding stage (*p* = 0.004) and sound type (*p* = 0.045; Table 3).

### 3.2 Hosts Approaching the Dummies During Sound Playback

Among all experimental points, there were a total of 14 points where hosts (the sum of ORW and BRW) approached the dummies during sound playback (*n* = 90). The number of points where ORW or BRW approached the dummies varied (Table 1). GLMM analysis results showed that when considering the hosts ORW and BRW separately, the number of points where individuals approached the dummies did not significantly differ between the breeding stages (host: *p* = 0.997, ORW: *p* = 0.998, BRW: *p* = 0.996). However, in terms of sound type, there were significant differences in the number of points where hosts or BRW approached the dummies when female CC sounds were played back compared to male CC sounds (host: *p =* 0.000, BRW: *p =* 0.004), while the difference was not significant for ORW (*p =* 0.997). Similarly, regardless of the early or late stages, ORW did not approach the dummies during the playback of male CC sounds. Moreover, in both breeding stages and sound types, the number of BRW approaching the dummies was greater than that of ORW (Table 2). From the GLMM analysis results (Table 3), there were no significant differences in the number of hosts (*p =* 0.998), ORW (*p =* 0.998), or BRW (*p =* 0.997) approaching the dummies between the breeding stages. In terms of hosts and BRW, there were significant differences in their numbers when female CC sounds were played back compared to male CC sounds (host: *p <* 0.001, BRW: *p* = 0.018), but no significant difference was observed for ORW (*p =* 0.997).

## 4 Discussion

Our study results show that playback of male CS calls did not elicit responses from CCs and their hosts. Although playback of both female and male CC calls attracted male CCs (no evidence of female CCs being attracted), female CC calls attracted more male CCs, and the attracted males approached more closely. Similarly, during the early stage, female and male CC calls attracted more male CCs than during the late stage, and the distance at which they were attracted during the early stage was smaller. Additionally, female CC calls attracted more hosts of CCs compared to male CC calls.

Both male and female CCs have evolved calls with sex‐specific characteristics (Moskát and Hauber 2021). In a short breeding season and competitive environment, effective recognition of acoustic signals is crucial for brood parasitic birds like CCs. In the experiment, playback of both female and male CC calls attracted male CCs, but female CC calls attracted more male CCs. Firstly, this could be because female CCs use their vocal signals to attract mates (Moskát and Hauber 2019; Xia et al. 2019; Hauber and Moskát 2025), which can also be considered territorial signals to conspecific females (Davies 2015; Moskát et al. 2020). Similarly, in other bird species, female vocalizations are used to attract mates (Langmore 1998). For instance, the vocalizations of the female Hainan partridge ( *Arborophila ardens* ) are effective at attracting conspecific individuals (Yang et al. 2011). CCs (both male and female) use their calls for territorial defense; however, CCs also tolerate neighbors with overlapping territories (Moskát et al. 2017). Birds often defend certain areas for resources, but their complex social functions can also reduce aggression among males (Hinde 1956). Therefore, our study further reveals the important role of female CC calls in communication between male and female CCs.

The range and mating system of CCs may depend on the intensity of competition with conspecific individuals, which could reduce their positive responses to other species (Davies 2000). For example, Moskát et al. (2017) found that CCs did not respond to playback of control sounds, which is consistent with our results showing that playback of male CS calls did not elicit behavioral responses from CCs. This may be an effective way for brood parasitic birds like the CC to reduce reproductive costs.

Female CC calls serve a deceptive function when it comes to hosts (Davies 2000; York and Davies 2017). For example, Marton et al. (2021), using a combination of sound playback and 3D models, demonstrated that female CC calls reduced the aggression of their host, the great reed warbler ( *Acrocephalus arundinaceus* ). Additionally, Wang et al. (2022) found that female CC calls were more likely to encourage hosts to leave their nests, similar to our results, where the playback of female CC calls was more effective at attracting CC hosts. This could be partly because female CCs are the ones primarily responsible for frequent nest visits and egg laying during brood parasitism. Therefore, their unique calls might serve to induce anti‐predator behaviors in hosts, reducing their resistance to parasitism (York 2021).

Moreover, the observed weak host response to male cuckoo calls may reflect adaptive desensitization through habituation. Given that male common cuckoos vocalize frequently throughout the breeding season (Moskát and Hauber 2022; Tryjanowski et al. 2024), hosts like Oriental reed warblers (ORW) and black‐browed reed warblers (BRW) may perceive these pervasive calls as lower‐risk stimuli. This habituation could conserve hosts' energy for more urgent threats, aligning with our finding that ORW entirely ignored male calls during playback (Table 2). In contrast, the rarer and acoustically distinct female “bubbling” calls (Deng et al. 2019) likely signal imminent parasitism risk, triggering heightened vigilance and approach behavior. Such differential responses optimize anti‐parasite defense: hosts habituate to predictable, low‐threat signals (male calls) while retaining sensitivity to cues directly linked to reproductive costs (female calls; Marton et al. 2021; York 2021).

While male calls are usually stereotypical, recent work indicates that syntactic errors, including syllable tripling, can occur and influence host responses (Tryjanowski, Jankowiak, Mikula, and Osiejuk 2025). However, only when male CCs sing at high frequencies can potential nesting hosts effectively identify cuckoos through grammatically abnormal songs (Tryjanowski, Jankowiak, Mikula, and Osiejuk 2025). Hence, our research suggests that hosts recognize the calls of female CCs as more threatening.

Our findings that hosts exhibited stronger responses to female cuckoo calls align with the emerging paradigm of sex‐specific threat assessment in brood parasite–host systems. Crucially, Tryjanowski, Jankowiak, Indykiewicz, et al. (2025) recently demonstrated through dummy experiments that host species (e.g., barn swallow *Hirundo rustica* ) attack female cuckoo models with significantly higher probabilities than male models, whereas non‐hosts show the opposite pattern. This parallel evidence from visual and acoustic domains jointly indicates that hosts have evolved fine‐tuned discrimination capabilities specifically targeting female cuckoos—the sex directly responsible for brood parasitism. The enhanced attractiveness of female calls to hosts in our playback experiments likely reflects an evolved adaptive vigilance mechanism, whereby hosts interpret these vocalizations as imminent threats to reproductive success (Marton et al. 2021; Tryjanowski, Jankowiak, Indykiewicz, et al. 2025). Such discrimination optimizes anti‐parasite defense efficiency: hosts can prioritize costly mobbing behaviors (Šulc et al. 2020) to the most dangerous parasite sex while reducing energy expenditure toward less threatening stimuli (e.g., male calls or non‐parasitic intruders). This functional interpretation resolves the apparent paradox wherein female calls simultaneously attract mates (intraspecific function) and provoke host defenses (interspecific function), underscoring the dual selective pressures shaping cuckoo vocal communication.

Furthermore, from the comparison of actual record data, the number of BRW (rare host of CCs) to approach the dummies was slightly more than that of ORW (main host of CCs). This aspect may stem from BRW's historical experience of parasitism by CC, as research indicates that the parasitic life history of some potential hosts prompts them to retain certain corresponding anti‐parasitic strategies (Peer and Sealy 2004). For instance, a host of CCs, the red‐billed leiothrix ( *Leiothrix lutea* ), was introduced to Hawaii more than 150 years ago, where the common cuckoo did not breed; however, even in the absence of parasitic pressure in Hawaii, it still maintains a strong egg rejection ability similar to its population in China (Yang et al. 2014). Medina and Langmore (2015) demonstrated that some potential host species, despite not being current hosts, still exhibit rejection rates as high as those observed in common primary hosts. This suggests that in our experiments, the attraction of BRW to the CC calls may be attributed to anti‐parasitic behaviors retained from past parasitism by CCs. In other words, while BRWs have largely evaded parasitism by CCs at present, they have retained certain anti‐parasitic strategies inherent to low‐cost behaviors. Therefore, these BRWs are willing to leave their nests to focus on CC calls far from their nests. On the contrary, the ORWs (main host of CCs) tend to preferentially protect their nests from being parasitized by CCs, rather than leaving their own nests to focus on CCs that are far away from their nests. Therefore, this may be one of the reasons why there are slightly fewer ORWs than BRWs to approach the dummies when playback CC calls occur. Additionally, it may also be that BRW's high level of “curiosity” leads to its willingness to venture closer to specimens or to playback female and male CC calls closer to its nest. However, the level of “curiosity” and the proximity of these sampling points to the nests of ORWs and BRWs was not objectively verified in our current experiment. It is necessary to address this oversight in future research.

While previous studies have shown that female cuckoo calls are known to attract males and manipulate hosts (Moskát and Hauber 2019; Marton et al. 2021; Jin et al. 2025), our findings reveal geographical and temporal specificity in these interactions. The heightened responsiveness (even though the data analysis was not significant) of black‐browed reed warblers—a rarely exploited host—suggests historical parasitism pressure may preserve anti‐parasite behaviors despite minimal current costs. Furthermore, the decline in male cuckoo attraction during late breeding highlights seasonal trade‐offs in territorial investment. Such nuances, quantified here in an understudied Asian system, emphasize that signal function is contingent on ecological context and host–parasite coevolutionary history.

In summary, our study found that the playback of female CC calls was more effective at attracting male CCs compared to the playback of male CC calls. Additionally, female calls were more effective at attracting CC hosts. Our research provides initial insights into the importance of female CC calls to communication between male and female CCs, with hosts recognizing the calls of female CCs as more threatening. Male CC calls play a crucial role in the intraspecific communication and territorial signaling of CCs, and hosts can recognize the calls of male CCs (Tryjanowski et al. 2024). Nevertheless, our research still has certain limitations, such as our daily testing times not always being in the morning (the peak period of bird activity), so tests conducted at midday or in the afternoon may have resulted in differences in observed response rates that were partly due to time effects rather than intrinsic differences between call types. A minimum of 200 m between playback points may not be sufficient to avoid spatial overlap—particularly in open habitats like reedbeds. Given the size of the home range of cuckoos, the same individuals may have responded at multiple sites. In addition, it is unclear whether cuckoos were present within hearing range before each playback. Without the presence of cuckoos, non‐responses may reflect absence rather than behavioral indifference, limiting the interpretability of the data. Further work is needed on different geographic populations of CCs and should also consider the potential influence of displaying different‐colored CC models when playing sounds, as well as investigating whether the behavior of CC nestlings differs in response to male and female calls.

## Author Contributions

**Hanlin Yan:** data curation (lead), formal analysis (lead), investigation (lead), methodology (equal), visualization (equal), writing – original draft (equal). **Wei Liang:** conceptualization (lead), funding acquisition (lead), supervision (lead), validation (equal), writing – review and editing (lead).

## Ethics Statement

The study was conducted in compliance with the law of China. Experimental procedures in China were in accordance with the Animal Research Ethics Committee of Hainan Provincial Education Centre for Ecology and Environment, Hainan Normal University (no. HNECEE‐2012‐003).

## Conflicts of Interest

The authors declare no conflicts of interest.

## Supporting information


**Audio S1.** Calls of the male Himalayan cuckoo for playback experiment.

**Audio S2.** Calls of the female common cuckoo for playback experiment.

**Audio S3.** Calls of the male common cuckoo for playback experiment.

## Data Availability

Data and song playback used for this study are provided as Supporting Information (Data Tables S1 and S2; Audios [Link], [Link] and Video S1) and can be found at https://figshare.com/s/66b24e39749388d90970 (DOI: 10.6084/m9.figshare.27222963).

## References

[ece372069-bib-0001] Bradbury, J. W. , and S. L. Vehrencamp . 2011. Principles of Animal Communication. Oxford University Press.

[ece372069-bib-0002] Briefer, E. , T. Aubin , K. Lehongre , and F. Rybak . 2008. “How to Identify Dear Enemies: The Group Signature in the Complex Song of the Skylark *Alauda arvensis* .” Journal of Experimental Biology 213: 317–326. 10.1242/jeb.013359 18203986

[ece372069-bib-0050] Catchpole, C. K. , and P. J. B. Slater . 2008. Bird Song: Biological Themes and Variations. 2nd ed. Cambridge University Press.

[ece372069-bib-0003] Davies, N. B. 2000. Cuckoos, Cowbirds and Other Cheats. T & AD Poyser.

[ece372069-bib-0051] Davies, N. B. 2015. Cuckoo: Cheating by Nature. Bloomsbury.

[ece372069-bib-0004] Deng, Z. , H. Lloyd , C. Xia , A. P. Møller , W. Liang , and Y. Zhang . 2019. “Components of Variation in Female Common Cuckoo Calls.” Behavioral Processes 158: 106–112. 10.1016/j.beproc.2018.10.007 30478018

[ece372069-bib-0005] Elek, Z. , M. Bán , A. Fülöp , A. Marton , M. E. Hauber , and C. Moskát . 2021. “Call Rate in Common Cuckoos Does Not Predict Body Size and Responses to Conspecific Playbacks.” Journal of Ornithology 162: 183–1192.

[ece372069-bib-0006] Hauber, M. E. , and C. Moskát . 2025. “Acoustic Overtones Improve the Discrimination of Conspecific Female Calls by Male Common Cuckoos From Similar Heterospecific Calls.” Animal Cognition 28: 45. 40471390 10.1007/s10071-025-01966-x PMC12141366

[ece372069-bib-0007] Hinde, A. 1956. “The Biological Significance of the Territories of Birds.” Ibis 98: 340–369.

[ece372069-bib-0008] Jiang, X. , C. Zhang , J. Liu , and W. Liang . 2021. “Female Cuckoo Calls Elicit Vigilance and Escape Responses From Wild Free‐Range Chickens.” Ethology Ecology & Evolution 33: 37–48.

[ece372069-bib-0009] Jin, S.‐J. , J.‐W. Lee , and J.‐H. Yoo . 2025. “The Structural Function of the Bubbling Call of the Female Common Cuckoo (*Cuculus canorus*).” Ibis. 10.1111/ibi.13412.

[ece372069-bib-0010] Langmore, N. E. 1998. “Functions of Duet and Solo Songs of Female Birds.” Trends in Ecology & Evolution 13: 136–140. 21238233 10.1016/s0169-5347(97)01241-x

[ece372069-bib-0011] Lei, F. , H. Zhao , A. Wang , Z. Yin , and R. B. Payne . 2005. “Vocalizations of the Common Cuckoo *Cuculus canorus* in China.” Acta Zoologica Sinica 51: 31–37.

[ece372069-bib-0013] Marler, P. 2004. “Bird Calls: A Cornucopia for Communication.” In Nature's Music. The Science of Birdsong, edited by P. Marler and H. Slabbekoorn , 132–177. Elsevier Science.

[ece372069-bib-0014] Marton, A. , A. Fülöp , M. Bán , M. E. Hauber , and C. Moskát . 2021. “Female Common Cuckoo Calls Dampen the Mobbing Intensity of Great Reed Warbler Hosts.” Ethology 127: 286–293.

[ece372069-bib-0015] Maynard‐Smith, J. , and D. Harper . 2003. Animal Signals. Oxford University Press.

[ece372069-bib-0058] Medina, I. , and N. E. Langmore . 2015. “The Costs of Avian Brood Parasitism Explain Variation in Egg Rejection Behaviour in Hosts.” Biology Letters 11: 20150296. 26156128 10.1098/rsbl.2015.0296 PMC4528443

[ece372069-bib-0016] Moskát, C. , Z. Elek , M. Bán , N. Geltsch , and M. E. Hauber . 2017. “Can Common Cuckoos Discriminate Between Neighbours and Strangers by Their Calls?” Animal Behaviour 126: 253–260.

[ece372069-bib-0017] Moskát, C. , and M. E. Hauber . 2019. “Sex‐Specific Responses to Simulated Territorial Intrusions in the Common Cuckoo: A Dual Function of Female Acoustic Signaling.” Behavioral Ecology and Sociobiology 73: 60.

[ece372069-bib-0018] Moskát, C. , and M. E. Hauber . 2021. “Male Common Cuckoos Use a Three‐Note Variant of Their “Cu‐Coo” Call for Duetting With Conspecific Females.” Behavioural Processes 191: 104472. 34363910 10.1016/j.beproc.2021.104472

[ece372069-bib-0019] Moskát, C. , and M. E. Hauber . 2022. “Quantitative Analysis of Vocalisation Types in Male Common Cuckoos' “Gowk” Call Complex.” Zoology 154: 126043. 36027693 10.1016/j.zool.2022.126043

[ece372069-bib-0020] Moskát, C. , and M. E. Hauber . 2023. “On the Sparrowhawk‐Like Calls of Female Common Cuckoos: Testing for Heterospecific Vocal Mimicry in a Conspecific Functional Context.” Behavioral Ecology and Sociobiology 77: 111.

[ece372069-bib-0021] Moskát, C. , M. E. Hauber , J. Růžičková , A. Marton , M. Bán , and Z. Elek . 2020. “Female–Female Aggression and Male Responses to the Two Colour Morphs of Female Common Cuckoos.” Science of Nature 107: 28. 10.1007/s00114-020-01680-3 PMC7306036 32564143

[ece372069-bib-0022] Osiejuk, T. S. 2014. “Differences in Frequency of Shared Song Types Enables Neighbour‐Stranger Discrimination in a Songbird Species With Small Song Repertoire.” Ethology 120: 893–903.

[ece372069-bib-0054] Peer, B. D. , and S. G. Sealy . 2004. “Fate of Grackle (*Quiscalus* spp.) Defenses in the Absence of Brood Parasitism: Implications for Long‐Term Parasite‐Host Coevolution.” Auk 121: 1172–1186.

[ece372069-bib-0023] Stoddard, P. K. 1996. “Vocal Recognition of Neighbors by Territorial Passerines.” In Ecology and Evolution of Acoustic Communication in Birds, edited by D. E. Kroodsma and E. H. Miller , 356–374. Cornell University Press.

[ece372069-bib-0052] Šulc, M. , G. Štětková , P. Prochazka , et al. 2020. “Caught on Camera: Circumstantial Evidence for Fatal Mobbing of an Avian Brood Parasite by a Host.” Journal of Vertebrate Biology 69: 1–6.

[ece372069-bib-0024] Trnka, A. , L. Ma , H. Yan , L. Wang , and W. Liang . 2023. “Defense Behavior of Two Closely Related but Geographically Distant Host Species Against Cuckoo Parasitism: A Next Test for the Parallel Coevolution.” Ecology and Evolution 13: e9931. 37304363 10.1002/ece3.10175 PMC10251422

[ece372069-bib-0025] Tryjanowski, P. , A. Golawski , Ł. Jankowiak , and A. P. Møller . 2024. “Reactions of Wintering Passerines to Male Calls of the European Cuckoo *Cuculus canorus* .” Scientifc Reports 14: 14204. 10.1038/s41598-024-64270-7 PMC11189894 38902276

[ece372069-bib-0026] Tryjanowski, P. , Ł. Jankowiak , P. Indykiewicz , F. Morelli , G. Grzywaczewski , and A. P. Møller . 2025. “Mobbing Behaviour of Hosts and Non‐Hosts Towards Cuckoo *Cuculus canorus* of Different Sex.” Acta Ethologica 28: 1–7.

[ece372069-bib-0027] Tryjanowski, P. , Ł. Jankowiak , P. Mikula , and T. S. Osiejuk . 2025. “Syntactically Aberrant Vocalization in Cuckoos Disrupts Communication but Triggers Host Responses.” Animal Behaviour 221: 123080.

[ece372069-bib-0028] Wang, J. , L. Ma , X. Chen , and C. Yang . 2022. “Female Cuckoo Calls Deceive Their Hosts by Evoking Nest‐Leaving Behavior: Variation Under Different Levels of Parasitism.” Animals 12: 1990. 35953979 10.3390/ani12151990 PMC9367515

[ece372069-bib-0029] Wang, Y. , M. Tian , J. Liu , X. Lu , A. P. Møller , and C. Xia . 2021. “Testing the Interspecific Function of Female Common Cuckoo “Bubbling” Call.” Frontiers in Ecology and Evolution 9: 725222.

[ece372069-bib-0030] Wyllie, I. 1981. The Cuckoo. Batsford.

[ece372069-bib-0031] Xia, C. , Z. Deng , H. Lloyd , A. P. Møller , X. Zhao , and Y. Zhang . 2019. “The Function of Three Main Call Types in Common Cuckoo Calls.” Ethology 125: 652–659.

[ece372069-bib-0032] Yang, C. , X. Chen , L. Wang , and W. Liang . 2022. “Defensive Adaptations to Cuckoo Parasitism in the Black‐Browed Reed Warbler ( *Acrocephalus bistrigiceps* ): Recognition and Mechanism.” Animal Cognition 25: 1299–1306. 35320446 10.1007/s10071-022-01613-9

[ece372069-bib-0057] Yang, C. , Y. Liu , L. Zeng , and W. Liang . 2014. “Egg Color Variation, but Not Egg Rejection Behavior, Changes in a Cuckoo Host Breeding in the Absence of Brood Parasitism.” Ecology and Evolution 4: 2239–2246. 25360264 10.1002/ece3.1096 PMC4201437

[ece372069-bib-0033] Yang, C. , L. Wang , W. Liang , and A. P. Møller . 2016. “Egg Recognition as Anti‐Parasitism Defence in Hosts Does Not Select for Laying of Matching Eggs in Parasitic Cuckoos.” Animal Behaviour 122: 177–181.

[ece372069-bib-0034] Yang, C. , L. Wang , W. Liang , and A. P. Møller . 2017. “How Cuckoos Find and Choose Host Nests for Parasitism.” Behavioral Ecology 28: 859–865.

[ece372069-bib-0035] Yang, C. , Y. Zhang , Y. Cai , B. G. Stokke , and W. Liang . 2011. “Female Crowing and Differential Responses to Simulated Conspecific Intrusion in Male and Female Hainan Partridge ( *Arborophila ardens* ).” Zoological Science 28: 249–253. 21466341 10.2108/zsj.28.249

[ece372069-bib-0036] Yoo, S. , H. N. Kim , J. W. Lee , and J. C. Yoo . 2020. “Seasonal and Diurnal Patterns of Population Vocal Activity in Avian Brood Parasites.” Ibis 162: 1001–1011.

[ece372069-bib-0037] York, J. E. 2021. “The Evolution of Predator Resemblance in Avian Brood Parasites.” Frontiers in Ecology and Evolution 9: 725842.

[ece372069-bib-0038] York, J. E. , and N. B. Davies . 2017. “Female Cuckoo Calls Misdirect Host Defences Towards the Wrong Enemy.” Nature Ecology and Evolution 1: 1520–1525. 29185512 10.1038/s41559-017-0279-3

[ece372069-bib-0039] Yu, J. , H. Lu , W. Sun , W. Liang , H. Wang , and A. P. Møller . 2019. “Heterospecific Alarm‐Call Recognition in Two Warbler Hosts of Common Cuckoos.” Animal Cognition 22: 1149–1157. 31506795 10.1007/s10071-019-01307-9 PMC6834739

[ece372069-bib-0040] Zhang, C. , X. Jiang , M. Li , J. Liang , J. Liu , and W. Liang . 2021. “Female Cuckoo Calls Elicit Anti‐Predatory Behavior in Birds.” Journal of Ethology 39: 393–398.

